# Tumor Progression Locus 2 Protects against Acute Respiratory Distress Syndrome in Influenza A Virus-Infected Mice

**DOI:** 10.1128/spectrum.01136-22

**Published:** 2022-08-18

**Authors:** Krishna Latha, Sanjana Rao, Kaori Sakamoto, Wendy T. Watford

**Affiliations:** a Department of Infectious Diseases, University of Georgia, Athens, Georgia, USA; b Department of Genetics, University of Georgia, Athens, Georgia, USA; c Department of Pathology, University of Georgia, Athens, Georgia, USA; Thomas Jefferson University

**Keywords:** influenza, Tpl2, ARDS, edema, biomarkers

## Abstract

Excessive inflammation in patients with severe influenza disease may lead to acute lung injury that results in acute respiratory distress syndrome (ARDS). ARDS is associated with alveolar damage and pulmonary edema that severely impair gas exchange, leading to hypoxia. With no existing FDA-approved treatment for ARDS, it is important to understand the factors that lead to virus-induced ARDS development to improve prevention, diagnosis, and treatment. We have previously shown that mice deficient in the serine-threonine mitogen-activated protein kinase, Tpl2 (MAP3K8 or COT), succumb to infection with a typically low-pathogenicity strain of influenza A virus (IAV; HKX31, H3N2 [x31]). The goal of the current study was to evaluate influenza A virus-infected *Tpl2^−/−^* mice clinically and histopathologically to gain insight into the disease mechanism. We hypothesized that *Tpl2^−/−^* mice succumb to IAV infection due to development of ARDS-like disease and pulmonary dysfunction. We observed prominent signs of alveolar septal necrosis, hyaline membranes, pleuritis, edema, and higher lactate dehydrogenase (LDH) levels in the lungs of IAV-infected *Tpl2^−/−^* mice compared to wild-type (WT) mice from 7 to 9 days postinfection (dpi). Notably, WT mice showed signs of regenerating epithelium, indicative of repair and recovery, that were reduced in *Tpl2^−/−^* mice. Furthermore, biomarkers associated with human ARDS cases were upregulated in *Tpl2^−/−^* mice at 7 dpi, demonstrating an ARDS-like phenotype in *Tpl2^−/−^* mice in response to IAV infection.

**IMPORTANCE** This study demonstrates the protective role of the serine-threonine mitogen-activated protein kinase, Tpl2, in influenza virus pathogenesis and reveals that host Tpl2 deficiency is sufficient to convert a low-pathogenicity influenza A virus infection into severe influenza disease that resembles ARDS, both histopathologically and transcriptionally. The IAV-infected *Tpl2^−/−^* mouse thereby represents a novel murine model for studying ARDS-like disease that could improve our understanding of this aggressive disease and assist in the design of better diagnostics and treatments.

## INTRODUCTION

Acute respiratory distress syndrome (ARDS) is a form of lung injury induced by a variety of insults, including sepsis, pneumonia, severe traumatic injury, and aspiration of gastric contents that lead to both hypoxia (lack of oxygen) and edema (fluid in the lung) (1, 2). Thirty to 40 percent of patients hospitalized for influenza develop bacterial pneumonia, which increases the likelihood of developing ARDS (3). Therefore, it is not surprising that the most common viral causative agent for ARDS is influenza A virus (4). Regardless of the underlying cause, ARDS is characterized by the acute onset of noncardiogenic pulmonary edema, leading to difficulty in breathing and acute hypoxemic respiratory failure (5). Patients who do survive typically have lasting impairments, including persistent pulmonary dysfunction, cognitive impairment, and muscle weakness (6, 7). Risk factors that lead to higher probability of developing ARDS include advanced age (8–10), female gender (11–13), and surgery (14, 15).

One barrier to ARDS management is the difficulty in its diagnosis. In an ARDS study canvassing 50 countries in which the physician diagnoses of ARDS were based on the Berlin definition (16), the clinician diagnosis of ARDS had an accuracy of less than 40%, with severe cases extending the probability of diagnosis to only 80% (17). Contributing to failure of ARDS diagnosis is the fact that the measurement of oxygen levels via ratio of arterial oxygen partial pressure to fractional inspired oxygen (PaO_2_/FiO_2_) is not always performed (18) or can vary on a patient-to-patient basis (19). While diagnosis of ARDS in its early stages is difficult, early detection allows for treatment with high-flow oxygen and optimal fluid management to avoid increased pulmonary edema, as well as pharmacological and antiviral therapies (20, 21). Considering that ARDS remains a difficult disease to diagnose and manage, it is vital to understand the disease etiology and progression to better prevent, diagnose, and treat it.

The general pathological features of ARDS typically involve three overlapping phases, (i) an inflammatory or exudative phase, (ii) a proliferative phase, and (iii) a fibrotic phase (22, 23). The exudative phase is marked with alveolar damage due to necrosis of the epithelium leading to accumulation of protein-rich edema fluid, along with signs of inflammation, including cytokine secretion and associated recruitment of leukocytes, especially neutrophils (5). The proliferative phase is marked by recovery of the epithelium by proliferation of type II pneumocytes along the basement membrane that secrete surfactant and ultimately differentiate into type I pneumocytes (23, 24). Moreover, this phase sees the transition from inflammatory cell-derived mediators of lung injury to anti-inflammatory macrophage- and fibroblast-derived growth factors that facilitate repair. The last phase is end-stage fibrosis, characterized by extensive fibrotic thickening of the interstitium, which compromises alveolar gas exchange and leads to hypoxia (2, 22). At this stage, the only treatment option is mechanical ventilation; however, recovery depends on many factors, including the level of care received and other treatments (25). Unfortunately, ARDS typically results in nearly 40% mortality, even with aggressive treatment (17, 26–29).

ARDS development post-influenza virus infection can be triggered by multiple mechanisms, including lung damage caused by viral cytopathic effects and inflammation-induced lung damage (30, 31), as well as secondary bacterial pneumonia (32, 33). Influenza virus-induced ARDS is generally associated with the secretion of the inflammatory cytokines interleukin 6 (IL-6), IL-10, granulocyte colony-stimulating factor (G-CSF), IL-1β, IL-8, monocyte chemoattractant protein 1 (MCP-1), and IL-12 (34–37), along with the recruitment and function of macrophages, neutrophils, and monocytes (38–42). However, it is often difficult to distinguish the initial cause of ARDS, and certain cases of ARDS have been bioinformatically assessed to be less inflammatory than others (43, 44). Moreover, ARDS can develop in patients with neutropenia (43, 44), and inflammatory cytokines do not always correlate with ARDS severity (45, 46). Therefore, it is hard to predict which cases of influenza virus-induced inflammation will progress to ARDS. Because early and aggressive intervention is critical in ARDS management, it is essential that we gain a better understanding of the host factors regulating inflammation and ARDS progression during influenza virus infection to better diagnose and treat it. One regulator of influenza virus-induced inflammation and pathogenesis is the serine-threonine kinase Tpl2 (47).

Tpl2 has previously been studied for its activation of extracellular signal-regulated kinase 1 and 2 (ERK1/2), Jun N-terminal protein kinase (JNK), and p38 in response to diverse Toll-like receptors (TLRs), cytokine receptors, G protein-coupled receptors, and Fc receptors (48). Tpl2 is constitutively associated with NF-κB p105 and A20-binding inhibitor of NF-κB 2 (ABIN-2) (49, 50), and following agonist stimulation, the inhibitor of kappa B kinase (IKK) complex phosphorylates NF-κB p105, triggering its K48-linked ubiquitination and proteosomal degradation (51). Tpl2 release from NF-κB p105 inhibition, coupled with its phosphorylation on serine 400 by IKK2 (52), enables Tpl2-dependent expression of various pro- and anti-inflammatory mediators. While Tpl2 has complex effects on cytokine regulation, the net effect of Tpl2 is believed to be promotion of inflammation due to Tpl2-dependent production of tumor necrosis factor (TNF), IL-1β, and COX2, among others (53–55). Consequently, Tpl2 inhibitors have been generated and are considered potential treatments for chronic autoimmunity (56).

More recently, Tpl2 has been recognized as an important regulator of antiviral responses by phosphorylating and altering the transcriptional activity of IRF3 (57). We previously demonstrated enhanced morbidity and mortality to influenza A virus (IAV) infection in *Tpl2^−/−^* mice with deteriorating clinical signs from 7 to 9 days postinfection (dpi) (47, 58). Live virus was undetectable in the lungs by 9 dpi, confirming viral clearance, albeit delayed, in the *Tpl2^−/−^* mice (47). Despite viral clearance, *Tpl2^−/−^* mice exhibited an excessive influx of inflammatory cells, specifically inflammatory monocytes and neutrophils, by 7 dpi. Tpl2 negatively regulates type I interferon (IFN) production in macrophages and dendritic cells in response to TLR stimulation (59). Consistent with this, we observed IFN-β overexpression in IAV-infected *Tpl2^−/−^* mice that positively correlated with upregulation of the chemokines, CCL2 and CXCL1, both of which also correlated with nitric oxide synthase 2 (NOS2) overexpression (47). Collectively, these data suggested that recruited inflammatory monocytes and neutrophils may contribute to severe influenza disease in *Tpl2^−/−^* mice; however, the precise cause of morbidity and mortality has not been determined. The goal of the current study was to evaluate IAV-infected *Tpl2^−/−^* mice clinically and histopathologically to gain insight into disease mechanisms, including the potential development of ARDS features and pulmonary insufficiency.

## RESULTS

### *Tpl2^−/−^* mice present with increased immunopathology upon influenza infection.

**FIG 1:**
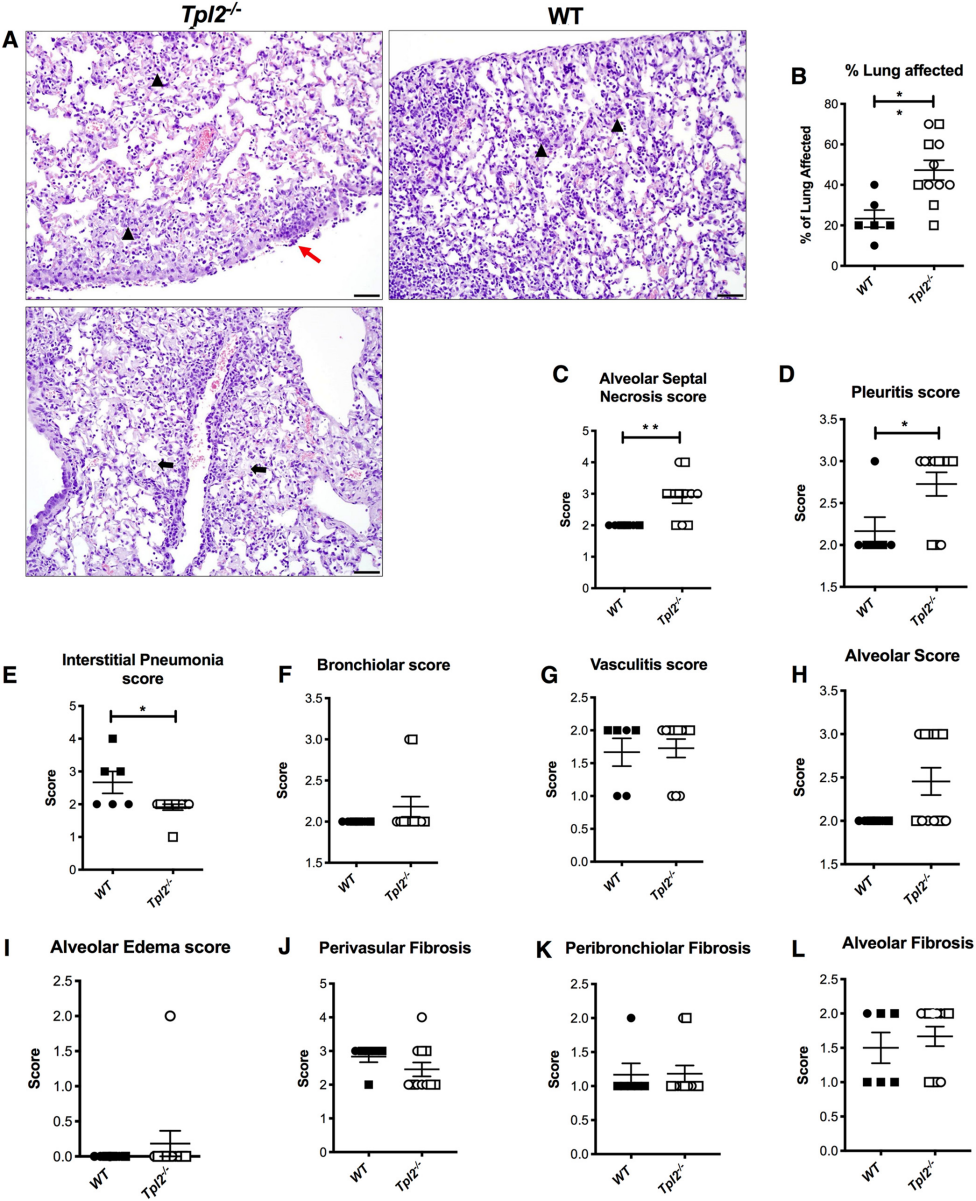
Increased severity and distribution of some pulmonary lesions in *Tpl2^−/−^* mice at 7 dpi with influenza A virus. WT (*n* = 6) and *Tpl2^−/−^* (*n* = 11) mice were infected intranasally with 10^4^ PFU of influenza A virus x31. At 7 dpi, the lungs were fixed in formalin, stained with H&E, and scored. (A) Representative images of *Tpl2^−/−^* (left) and WT (right) lungs at ×20 magnification highlight the pleuritis, alveolar septal damage, and interstitial pneumonia. Black arrows indicate alveolar septal necrosis, black arrowheads indicate interstitial pneumonia, and red arrows indicate pleuritis. (B to I) Pooled scores for all lungs in the two groups. (J to L) Separate sections of the same lungs were stained with Masson’s trichrome stain and scored for fibrosis. Squares represent male mice, and circles represent female mice. Unpaired Student’s *t* test; *, *P < *0.05; **, *P < *0.01. Data are representative of 2 experiments.

IAV-infected *Tpl2^−/−^* mice show severe inflammation, as measured by cytokine expression, by 7 dpi (47). To gain further insight into the etiology of disease in IAV-infected *Tpl2^−/−^* mice, we assessed the lung tissue for pathological alterations at 7 dpi because this was the clinically divergent time point at which wild-type (WT) mice show signs of recovery, whereas *Tpl2^−/−^* mice become progressively worse (47). Histopathology of the lungs was scored by a board-certified veterinary pathologist blinded to the groups according to the scale defined in Materials and Methods. Lung sections from *Tpl2^−/−^* mice showed a greater percentage of the lung area affected by inflammation at 7 dpi (Fig. 1B). Increased alveolar septal necrosis and inflammation of the pleura were also observed in *Tpl2^−/−^* lung sections (Fig. 1A, C, and D), indicating that increased interstitial and alveolar damage extended to the pleura in *Tpl2^−/−^* mice. While interstitial pneumonia was higher in WT mice, other indices, including bronchiolar, vasculitis, alveolar, and alveolar edema scores, were similar between WT and *Tpl2^−/−^* mice at 7 dpi (Fig. 1E to I). We also examined fibrosis using Masson’s trichrome staining and found no difference in fibrosis at 7 dpi between WT or *Tpl2^−/−^* mice (Fig. 1J to L).

**FIG 2:**
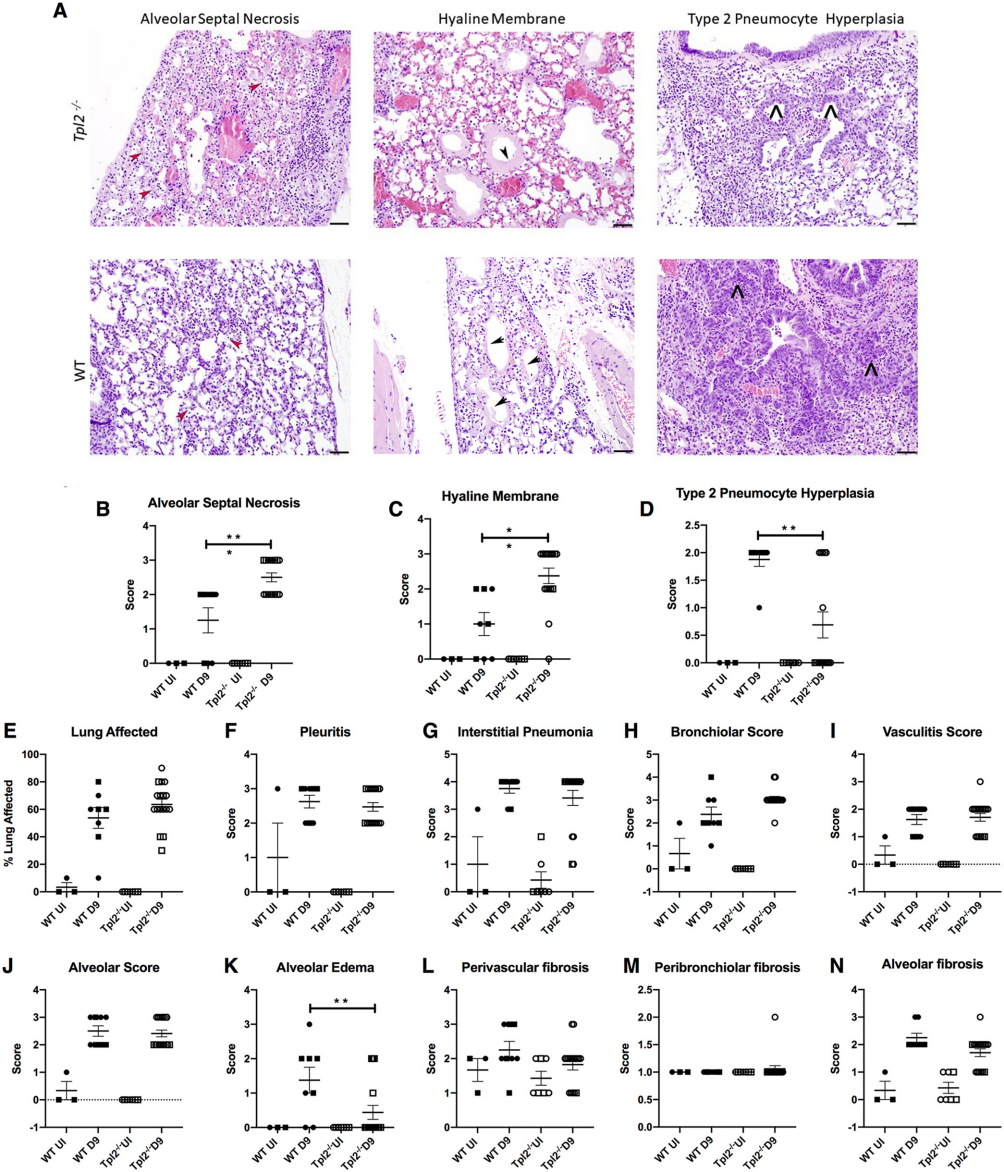
Further increase in histopathologic damage in the *Tpl2^−/−^* mice at 9 dpi. WT (*n* = 8) and *Tpl2^−/−^* (*n* = 16) mice were infected intranasally with 10^4^ PFU of influenza A virus x31 with uninfected controls for WT (*n* = 3) and *Tpl2^−/−^* (*n* = 7). At 9 dpi, the lungs were fixed in formalin, stained with H&E, and scored. (A) Representative images of *Tpl2^−/−^* (top) and WT (bottom) lungs at ×20 magnification highlight the alveolar septal necrosis (ASN), hyaline membrane, and type II pneumocyte hyperplasia (T2PH). Red arrowheads indicate ASN, black arrowheads indicate hyaline membranes, and black carets indicate T2PH. (B to K) Pooled scores for all lungs in the two groups. (L to N) Additional sections of the same lungs were stained with Masson’s trichrome and scored for fibrosis. Squares represent male mice, and circles represent female mice. Data are representative of 2 experiments. One-way ANOVA with Tukey’s multiple-comparison test was performed. *, *P < *0.05; **, *P < *0.01; ***, *P < *0.001.

Since *Tpl2^−/−^* mice succumb to the infection at approximately 10 dpi (47), we further examined both WT and *Tpl2^−/−^* mice at 9 dpi, including uninfected mice for baseline comparison. At this time point, prominent infection-induced increases were noted in both WT and *Tpl2^−/−^* mice for nearly every parameter assessed except perivascular and peribronchiolar fibrosis scores (Fig. 2). Significantly more severe alveolar septal necrosis and hyaline membrane formation (fibrin lining the septa) (Fig. 2A to C) were noted in *Tpl2^−/−^* mice than in WT mice. Notably, these histopathologic lesions are seen in patients with pathogenic influenza infection or influenza-induced ARDS (60–62). The representative images that depict alveolar septal necrosis as well as the hyaline membranes in *Tpl2^−/−^* lung sections are from mice that naturally succumbed to the infection. However, similar lesions were also observed in severely ill mice that were humanely euthanized. Conversely, type 2 pneumocyte hyperplasia (T2PH), visualized by the presence of greater numbers of larger, more basophilic cells lining the alveoli, was significantly higher in WT than in *Tpl2^−/−^* mice at 9 dpi (Fig. 2A and D). Increased T2PH in WT mice indicates regeneration of the epithelium postinjury and is characteristic of influenza recovery (60, 63). The percentages of lung affected and pleuritis had increased sharply from their values observed at 7 dpi, especially in WT mice, and were no longer statistically different at 9 dpi (Fig. 2E and F), nor were several other measures (Fig. 2G through J). Unexpectedly, by histological examination, alveolar edema appeared to be decreased in lungs of *Tpl2^−/−^* mice compared to WT mice at 9 dpi (Fig. 2K). We still did not observe significant differences in fibrosis by histochemistry at 9 dpi (Fig. 2L to N).

### Diagnostic profiling reveals elevated lactate dehydrogenase levels and pulmonary edema in IAV-infected *Tpl2^−/−^* mice, indicative of acute lung injury.

**FIG 3:**
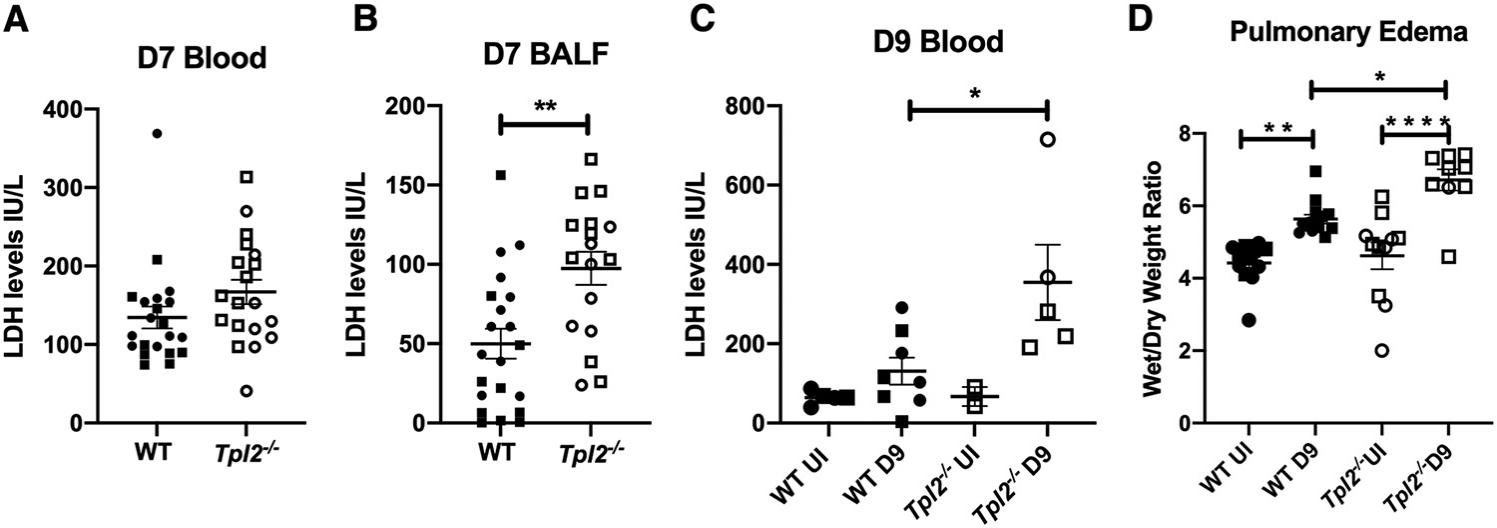
Increased lung injury and edema are observed in *Tpl2^−/−^* mice at 9 dpi. (A and B) WT (*n* = 21) and *Tpl2^−/−^* (*n* = 19) mice were infected intranasally with 10^4^ PFU of influenza A virus x31 for 7 days. Blood was collected by cardiac puncture (A), and BALF was collected by intratracheal instillation twice with the same 1 mL of PBS (B). LDH was measured as described in Materials and Methods. Data are representative of 2 experiments. Unpaired Student’s *t* test; *, *P < *0.05; **, *P < *0.01; ***, *P < *0.001. Squares represent male mice, and circles represent female mice. (C) WT (*n* = 8) and *Tpl2^−/−^* (*n* = 5) mice were intranasally infected with 10^4^ PFU of influenza A virus x31 for 9 days with uninfected controls for WT (*n* = 5) and *Tpl2^−/−^* (*n* = 2). At 9 dpi, blood was collected by cardiac puncture. LDH release was assayed as in panel B. (D) WT (*n* = 14) and *Tpl2^−/−^* (*n* = 9) mice were infected intranasally with 10^4^ PFU of influenza A virus x31 with uninfected controls for WT (*n* = 12) and *Tpl2^−/−^* (*n* = 11). At 9 dpi, the lungs were collected, weighed, and dried for 7 days at 50°C before weighing again to calculate the pulmonary edema. One-way ANOVA with Tukey’s multiple-comparison test was performed; *, *P < *0.05; **, *P < *0.01; ***, *P < *0.001. Squares represent male mice; circles represent female mice.

Lactate dehydrogenase (LDH) is an abundant intracellular enzyme that converts pyruvate to lactate in the presence of NADH. LDH levels in blood are normally low, but tissue damage can cause its release into the circulation. Elevated systemic LDH level is a widely accepted method to evaluate cellular damage during various pathological conditions, including IAV-induced apoptosis and COVID-19 severity prognosis (64–66). We first examined LDH levels in the blood and bronchoalveolar lavage fluid (BALF) at 7 dpi and found that, while *Tpl2^−/−^* mice did not show a statistically significant increase in circulating LDH levels in blood at this time point (Fig. 3A), they did exhibit increased LDH release locally in the BALF (Fig. 3B). By 9 dpi, elevated LDH release was also observed systemically in the blood of *Tpl2^−/−^* mice (Fig. 3C), consistent with progression of the disease by that time point. Pulmonary edema is a major component of ARDS and is often used to clinically define the condition (67, 68). Therefore, we examined pulmonary edema by measuring the wet/dry lung weights at 9 dpi. *Tpl2^−/−^* mice had significantly higher levels of pulmonary edema than WT mice at 9 dpi (Fig. 3D), consistent with increased morbidity in *Tpl2^−/−^* mice (47).

**FIG 4:**
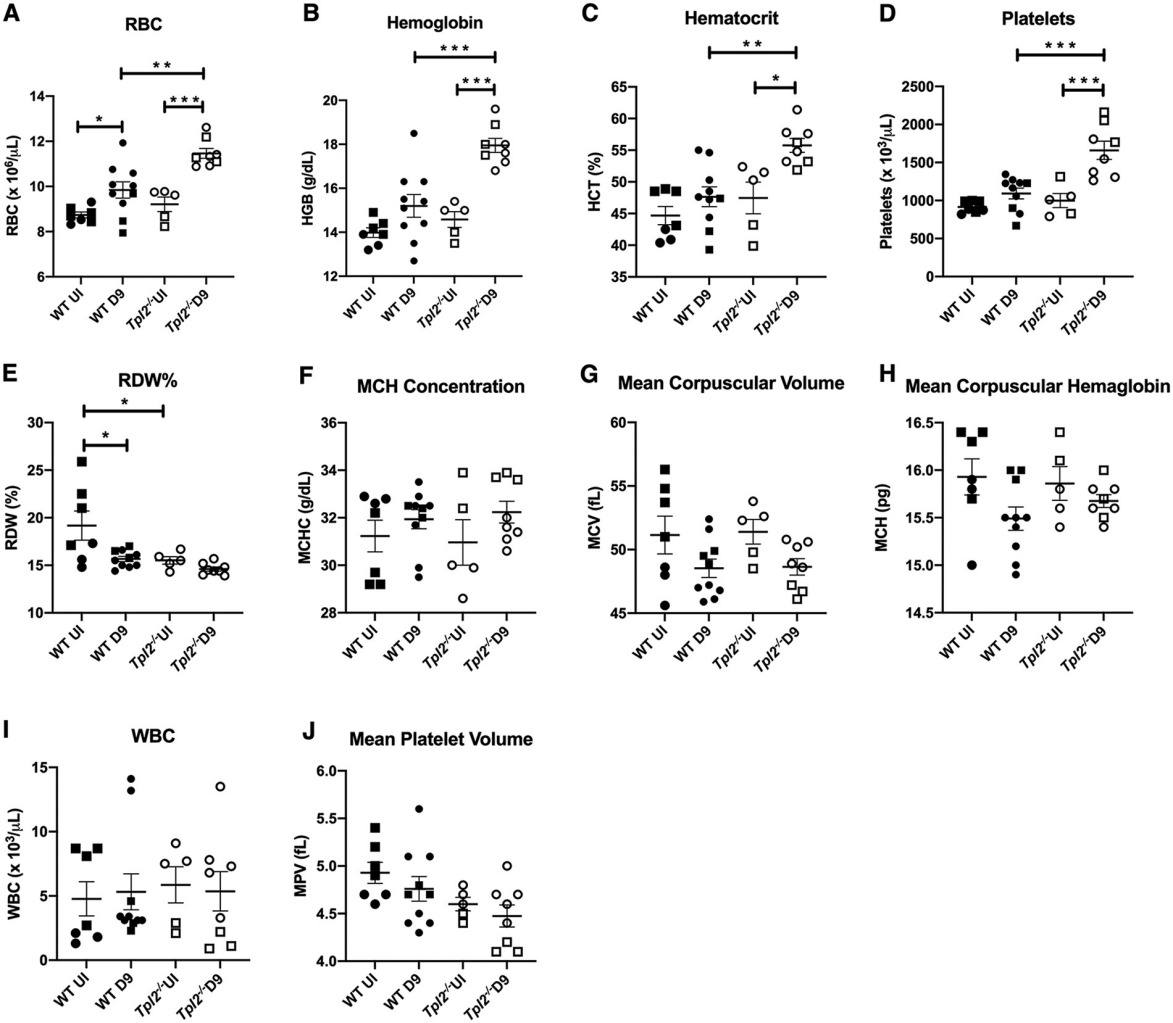
Red blood cell analysis in *Tpl2^−/−^* compared to WT mice at 9 dpi. (A to J) WT (*n* = 10) and *Tpl2^−/−^* (*n* = 8) mice were infected intranasally with 10^4^ PFU of influenza A virus x31 with uninfected controls for WT (*n* = 7) and *Tpl2^−/−^* (*n* = 5). At 9 dpi, blood was collected from the mice by cardiac puncture and assessed for red blood cell indices. RBC, red blood cells; HGB, hemoglobin; HCT, hematocrit; RDW%, percent red cell distribution width; MCV, mean corpuscular volume; MCH, mean corpuscular hemoglobin; MCHC, mean corpuscular hemoglobin concentration; MPV, mean platelet volume. Data are representative of 2 experiments. Squares represent male mice, and circles represent female mice. One-way ANOVA with Tukey’s multiple-comparison test was performed. *, *P < *0.05; **, *P < *0.01; ***, *P < *0.001.

We next examined the complete blood profile for systemic alterations at 9 dpi. Higher red blood cell (RBC) counts, hemoglobin (HgB) content, hematocrit (HCT), and platelets were observed in circulation in *Tpl2^−/−^* mice compared to WT at 9 dpi (Fig. 4A to D). However, no differences were observed for various other measures, including red cell distribution width (RDW), mean corpuscular hemoglobin concentration (MCHC), mean corpuscular volume (MCV), and mean corpuscular hemoglobin (MCH), which are primarily measured to assess anemia (Fig. 4E to H). There was also no difference in white blood cell (WBC) counts or mean platelet volume (MPV) (Fig. 4I and J). Consistent with increased pulmonary edema in IAV-infected *Tpl2^−/−^* mice noted above, increased RBC, HgB, and HCT values in the *Tpl2^−/−^* mice could represent secondary effects of dehydration due to loss of fluid into the alveolar compartment.

### ARDS exudative biomarkers are increased in IAV-infected *Tpl2^−/−^* mice.

An intense pulmonary neutrophilic host response is one of the most prominent causative hallmarks for ARDS development (69–72). In mouse models of IAV-induced ARDS, neutrophils have been shown to contribute to lung damage, with clinical signs of weight loss and morbidity peaking at approximately 6 dpi (73–75). Similarly, we have previously noted excessive influx of neutrophils and monocytes into the lungs of IAV-infected *Tpl2^−/−^* mice coincident with NOS2 overexpression at 7 dpi. Peak morbidity is observed shortly thereafter at approximately 9 to 10 dpi (47). Considering the epithelial damage observed histopathologically in *Tpl2^−/−^* mice at 7 and 9 dpi, along with inflammation noted in IAV-infected *Tpl2^−/−^* mice previously (47) and the similar clinical course and outcomes seen in the exudative phase of ARDS (5, 23, 76), we next examined the expression of early ARDS biomarkers in IAV-infected WT and *Tpl2^−/−^* mice.

**FIG 5:**
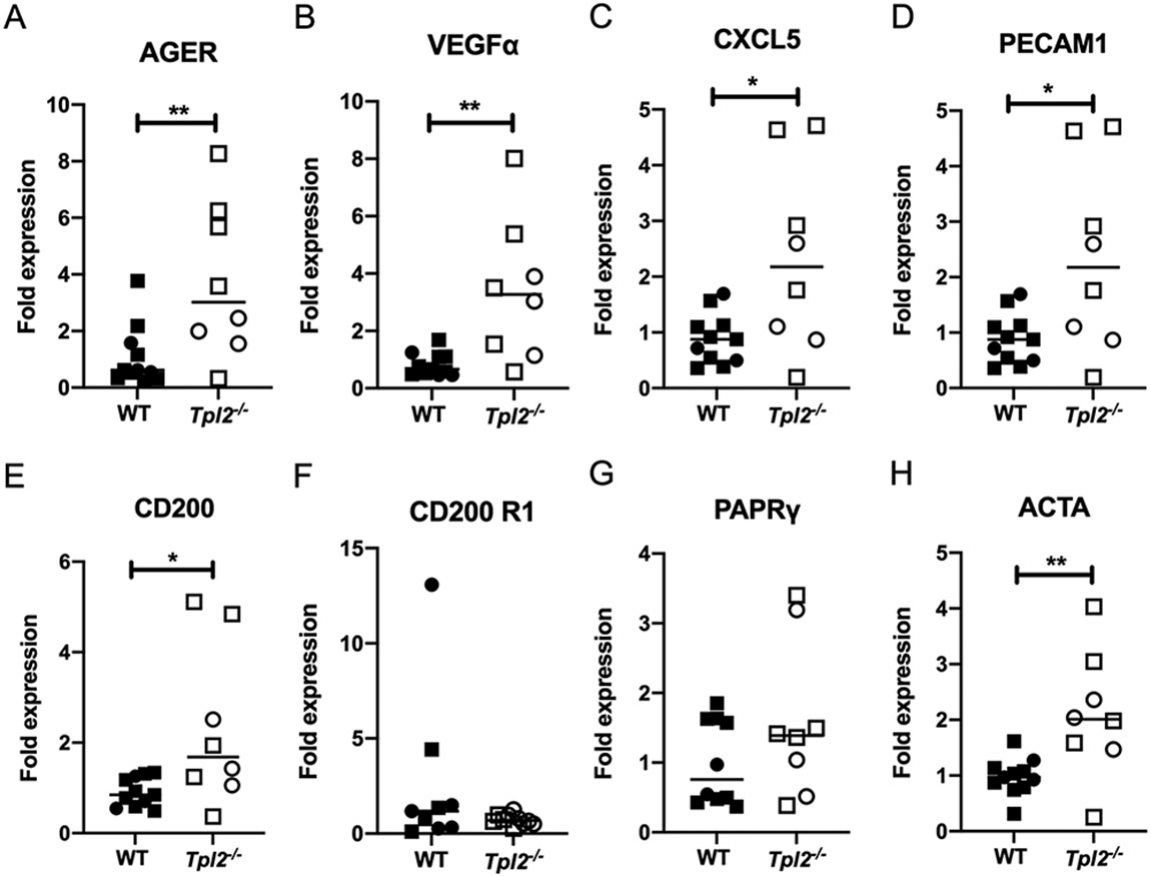
ARDS genes are overexpressed in lungs of IAV-infected *Tpl2*^−/−^ mice at 7 dpi. WT (*n* = 11) and *Tpl2^−/−^* (*n* = 8) mice were infected intranasally with 10^4^ PFU of influenza A virus x31 for 7 days. Lungs were harvested and homogenized for RNA extraction for gene expression analysis by real-time quantitative reverse transcription-PCR (qRT-PCR). Data are representative of 3 experiments. Squares represent male mice, and circles represent female mice. Unpaired Student’s *t* test; *, *P < *0.05; **, *P < *0.01.

Whole lungs were analyzed for expression of ARDS exudative-phase biomarkers at 7 dpi, which corresponds to the earliest clinically divergent time point between IAV-infected WT and *Tpl2^−/−^* mice. This is also the time point that exudative monocytes and neutrophils were significantly increased in IAV-infected *Tpl2^−/−^* mice (47). Since ARDS manifests due to issues involving the epithelium, endothelium, and other contributing cell types like alveolar macrophages, broadly representative biomarkers were selected. We examined several biomarkers of the exudative phase of ARDS, including receptor for advanced glycosylation end products (RAGE), vascular endothelial growth factor (VEGFα), CXCL5 (or ENA78, which has structural homology to IL-8), and platelet and endothelial cell adhesion molecule 1 (PECAM-1) (76–79). RAGE is expressed in epithelial cells, whereas VEGFα, PECAM-1, and CXCL5 are associated with endothelial activation during ARDS (80), linked with the restructuring of the endothelium to facilitate neutrophil adhesion (81–83) and angiogenesis (84–88). mRNA expression of RAGE (encoded by *Ager*), VEGFα, CXCL5, and PECAM-1 were upregulated in lungs of *Tpl2^−/−^* mice compared to WT mice (Fig. 5A to D).

During influenza virus infection, CD200 receptor (CD200R) expression is upregulated on various myeloid cells, especially alveolar macrophages. Concomitantly, its ligand, CD200, is expressed by thymocytes, dendritic cells, activated T cells, resting B cells, and endothelial and epithelial cells (89, 90). CD200 interaction with CD200R suppresses the inflammatory activity of alveolar macrophages (91). Consequently, CD200*^−/−^* mice developed ARDS in response to influenza virus infection due to increased proinflammatory macrophage function (91). Furthermore, intratracheal instillation of lipopolysaccharide (LPS) reduced CD200 expression in mice in a model of LPS-induced lung injury. Therefore, reduced expression of CD200 has been associated with mouse models of ARDS via its reduced ability to suppress the inflammatory function of alveolar macrophages (91, 92). Notably, we observed elevated levels of CD200 (Fig. 5E); however, no difference in the expression of CD200R1 was observed in lungs of IAV-infected *Tpl2^−/−^* mice at 7 dpi (Fig. 5F).

We also examined another biomarker, peroxisome proliferator-activated receptor gamma (PPAR-γ), to represent the interaction between the pulmonary epithelium and alveolar macrophages (76). PPAR-γ is a nuclear receptor expressed by alveolar macrophages that mediates anti-inflammatory effects by suppressing inflammatory products, like MCP-1, MIP2, NOS2, COX2, intercellular adhesion molecule (ICAM), and P-selectin, as well as preventing lung injury and edema (93, 94). PPAR-γ trended higher in *Tpl2^−/−^* lungs at 7 dpi, but this did not reach statistical significance (Fig. 5G). Finally, actin alpha 2 (ACTA), which is required for the movement of myofibroblasts in the early stages of the lung injury and is upregulated in ARDS patients (95), was overexpressed in *Tpl2^−/−^* lungs at 7 dpi (Fig. 5H). Collectively, we observed overexpression of AGER, VEGFα, CXCL5, PECAM-1, and ACTA in IAV-infected *Tpl2^−/−^* mice at 7 dpi, which corresponds to ARDS biomarker profiles in human patients. No significant differences in CD200R1 or PPAR-γ expression, along with increased expression of CD200, were observed in lungs of IAV-infected *Tpl2^−/−^* mice. Collectively, these data suggest that influenza virus infection induces significantly increased acute lung injury in *Tpl2^−/−^* mice, which express an ARDS-like histopathologic and transcriptional phenotype.

## DISCUSSION

Our previous study established hypercytokinemia and increased pulmonary recruitment of inflammatory monocytes and neutrophils in IAV-infected *Tpl2^−/−^* mice at 7 dpi, at which time they started showing increased weight loss and clinical scores compared to WT mice. Accordingly, *Tpl2^−/−^* mice succumbed to IAV infection by 10 dpi (47). In this study, detailed examination of lung histopathology from 7 to 9 dpi revealed alveolar septal necrosis at 7 dpi that became more prominent in the *Tpl2^−/−^* mice by 9 dpi with formation of hyaline membranes, both of which are characteristic of ARDS development. Conversely, WT mice showed signs of regenerating epithelium, as assessed by type 2 pneumocyte hyperplasia, by 9 dpi, consistent with attempts at recovery that were reduced in the absence of Tpl2. We also observed pleuritis and higher levels of LDH in the BALF of *Tpl2^−/−^* mice at 7 dpi and in the blood at 9 dpi. Notably, increased morbidity in the *Tpl2^−/−^* mice was accompanied by increased pulmonary edema, another hallmark of ARDS, at 9 dpi. Assessment of ARDS exudative phase biomarkers showed that *Tpl2^−/−^* mice display increased expression of RAGE, VEGFα, PECAM-1, CXCL5, and ACTA at 7 dpi. Therefore, the severe pathology in IAV-infected *Tpl2^−/−^* mice resembles ARDS both histopathologically and transcriptionally.

The early stages of influenza-related acute alveolar injury are characterized by denudation of the alveolar epithelium that then progresses to widening of the alveolar septa due to fluid leakage from the vasculature, which, along with fibrin thrombi and associated ischemia, might be the cause for alveolar septal necrosis. The last stage of viral pneumonia is re-epithelialization of the alveolar septa and infiltration by leukocytes (60, 96). Moreover, IAV-induced changes to the bronchioles (necrotizing bronchiolitis) are not long-lasting and are mainly seen subacutely as thickened epithelial linings, primarily due to epithelial regeneration and bronchial inflammation (96). Review of the histology observed in various influenza patient lungs postmortem showed that cases involving alveolitis (alveolar inflammation caused by damage to the alveolar surfaces) were devoid of virus detection but showed regeneration of the epithelium within 5 dpi (60). In *Tpl2^−/−^* mice, we observed a higher occurrence of alveolar septal necrosis at 7 dpi, which became more prominent by 9 dpi. This was coupled with formation of hyaline membranes despite undetectable virus by 9 dpi (47). These findings confirm that the pathology seen in *Tpl2^−/−^* mice results from a dysregulated immune response rather than uncontrolled viral replication.

Alveolar septal necrosis observed by 7 dpi is consistent with the diffuse alveolar damage characteristic of the early stages of ARDS (61, 62). The biomarkers overexpressed at 7 dpi, including AGER, VEGFα, CXCL5, PECAM-1, and ACTA, are representative of the early exudative phase of ARDS. CD200 is also an early phase marker of ARDS development; decreased expression has been shown to lead to ARDS development due to impaired immunoregulation. However, we noted instead that CD200 expression is increased in IAV-infected *Tpl2^−/−^* mice. CD200 is transcriptionally induced by inflammatory cytokines IFN-γ and TNF via STAT1, IRF1, and NF-κB (97). Because IFN-γ protein levels are highly upregulated in the lungs of IAV-infected *Tpl2^−/−^* compared to WT mice (47), increased CD200 expression in the lungs of *Tpl2^−/−^* mice could reflect the ongoing intense inflammation that ultimately contributes to acute lung injury and ARDS-like disease.

Pulmonary edema has been associated with the endogenous activity of nitric oxide synthase 2 (NOS2) in various models and clinical cases of lung injury, including hypercalcemia, endotoxin treatment, influenza, and ARDS (98–101). Additionally, NOS2 was found to be required for pathological vascular changes in the lungs (102). Indeed, we have observed increased NOS2 expression in the *Tpl2^−/−^* mice at 7 dpi (47). The development of pulmonary edema is consistent with the dyspnea observed in *Tpl2^−/−^* mice late during the disease course and ultimately explains the morbidity and mortality observed in *Tpl2^−/−^* mice via an ARDS-like mechanism. While reduced alveolar edema was noted in the *Tpl2^−/−^* mice by histology, this could be due to random sampling of less affected areas by histology. However, whole-organ analysis by quantitation of lung wet/dry weight ratio clearly showed a significant increase in pulmonary edema in IAV-infected *Tpl2^−/−^* mice. The pulmonary edema observed by weight could potentially explain the increased RBC, HgB, and HCT values in the *Tpl2^−/−^* mice secondary to dehydration, due in part to loss of fluid into the alveolar compartment. Poor oxygenation could also be driving upregulation of RBC production in the *Tpl2^−/−^* mice. Increased platelet counts in the *Tpl2^−/−^* mice are consistent with the increased inflammation observed in this group. Furthermore, alveolar damage seen from 7 to 9 dpi by histologic examination has been associated with excessive inflammation mediated by inflammatory cells, like neutrophils (62), in cases of ARDS caused by influenza and COVID-19 (63, 102, 103). Thus, Tpl2 ablation causes hypercytokinemia, inducing the excessive cellular influx that most likely induces the damage to the alveolar septa.

In studies examining the role of Tpl2 in lung injury induced by mechanical ventilation, either genetic ablation or pharmacological inhibition of Tpl2 before and after the ventilation reduced the severity of acute lung injury (104). In contrast, another study reported that Tpl2 kinase genetic inhibition was unable to prevent ventilation-induced lung injury (105). Our study is the first to show that Tpl2 serves a protective role during IAV-induced lung injury by preventing severe inflammation and ARDS development. The precise role of Tpl2 in acute lung injury is likely to be context dependent, with its protective role in virus-induced lung inflammation associated with its negative regulation of type I IFNs. Future studies will seek to further dissect the Tpl2-dependent host response to influenza virus infection within the different cellular compartments, including the epithelium, endothelium, and alveolar macrophages requisite for severe influenza disease development and ARDS progression.

In 2011, a committee established by the American Thoracic Society (ATS) proposed a list of features used to define ARDS in animals (106), encompassing (i) histological evidence of tissue injury, (ii) alterations in the alveolar-capillary barrier, (iii) an inflammatory response, and (iv) lung physiological dysfunction. Three of the four criteria should be met to diagnose ARDS in an animal model. Herein, we provide histological evidence of acute lung injury as well as pulmonary edema in IAV-infected *Tpl2^−/−^* mice compared to WT mice. Furthermore, our previous study demonstrated an enhanced inflammatory response and increased pulmonary recruitment of inflammatory monocytes and neutrophils (47). Collectively, these data establish IAV-infected *Tpl2^−/−^* mice as a novel murine model for studying ARDS-like disease. One advantage of the IAV-infected *Tpl2^−/−^* mouse model is the ability to elicit severe pulmonary disruption to a normally low-pathogenicity virus. This may more accurately reflect how the immune status predisposes some individuals to severe disease in response to even low-pathogenicity seasonal influenza. Furthermore, this is a clinically relevant model because influenza is the leading cause of virus-induced ARDS. Importantly, this model is still being characterized, and other potential effects of Tpl2 deficiency on the host response to influenza virus may also contribute to ARDS development. Ultimately, knowledge gained from studying ARDS development in response to severe influenza in mice could improve our understanding of this aggressive disease and assist in the design of better diagnostics and treatments.

## MATERIALS AND METHODS

### Mice and viruses.

Wild-type (WT) C57BL/6 mice were purchased from Jackson Laboratory. *Tpl2^−/−^* mice, backcrossed 10 generations onto the C57BL/6 strain, were kindly provided by Philip Tsichlis (47). Animals were housed in microisolator cages in the Central Animal Facility of the University of Georgia (UGA) College of Veterinary Medicine. All animal experiments were performed in accordance with the guidelines provided by the Guide for Care and Use of Laboratory Animals. The Institutional Animal Care and Use Committee (IACUC) of the University of Georgia approved all animal experiments.

Embryonated specific-pathogen-free (SPF) eggs were purchased from the Poultry Diagnostics and Research Center, UGA. Influenza A virus (IAV; HKX31, H3N2; here referred to as x31) stocks were kindly provided by Mark Tompkins (University of Georgia). The virus was propagated in the allantoic cavity of 9- to 11-day-old, embryonated SPF chicken eggs at 37°C for 72 h, and viral titers were enumerated by plaque assays as described (47).

### Influenza virus infection of mice.

Age-matched 6- to 8-week-old WT and *Tpl2^−/−^* mice were anesthetized with 250 mg/kg of 2% (wt/vol) avertin (2,2,2-tribromoethanol; Sigma), followed by intranasal instillation of 50 μL phosphate-buffered saline (PBS) containing 10^4^ PFU of influenza A virus (x31). The mice were maintained postinfection as described previously (47). Mice used for lung analysis were euthanized by intraperitoneal administration of approximately 800 mg/kg of 2% (wt/vol) avertin. Death was confirmed by exsanguination or cervical dislocation. Mice used solely for blood collection for complete blood count (CBC) analysis were euthanized by CO_2_ asphyxiation, and death was confirmed by cervical dislocation.

### Analysis of gene expression.

At 7 and 9 dpi, pulmonary gene expression was assessed by real-time PCR as described (47). The following probes were used: *Acta* (Mm01546133), *Ager* (Mm001134790), *Cd200* (Mm00487740), *Cd200r1* (Mm00491164), *Cxcl5* (Mm00436451), *Pparγ* (Mm00440940), *Pecam1* (Mm 01242584), *Ptges2* (Mm00460181), and *Vegfα* (Mm00437306). β-Actin endogenous control was used as a housekeeping gene for normalization using the threshold cycle (ΔΔ*C_T_*) method (107). The sequences are as follows: β-actin forward primer, CGATGAAGATCAAG/ATCATTGC; β-actin reverse primer, AAGCATTTGCGGTGGAC; and β-actin probe, TCCACCTTCCAGCAGATGTGGATCAGCAAG.

### Histology.

Mice were uninfected or infected with 10^4^ PFU of IAV (x31) for 7 or 9 days. Lungs were harvested and fixed with 10% neutral buffered formalin and processed into 4-μm-thick sections for hematoxylin and eosin (H&E) staining. The sections were scored by a board-certified veterinary pathologist in a blinded manner according to the following criteria:

- Alveolar/alveolar edema/pleuritis scores: focal lesion, 1 point; multifocal lesions, 2 points; multifocal to coalescing lesions, 3 points; the majority of lobule affected, 4 points.
- Interstitial pneumonia score: alveolar septa infiltrated and thickened by 1 leukocyte layer, 1 point; 2-cell-thick-layer infiltration of alveolar septa, 2 points; 3-cell-thick-layer infiltration of alveolar septa, 3 points; 4-cell-thick-layer infiltration of alveolar septa, 4 points.
- Bronchiolar score: focally affected bronchiole, 1 point; multifocally affected bronchioles, 2 points; the majority of the bronchioles in a lobule affected, 3 points; bronchioles diffusely affected in a lobule, 4 points.
- Vasculitis score: infiltration of vessel wall by leukocytes, 1 point; infiltration and separation of smooth muscle cells in the vessel wall, 2 points; infiltration and fibrinoid change, 3 points.

### Measurement of pulmonary edema.

Infected WT and *Tpl2^−/−^* mice were sacrificed at 9 dpi, and their lungs were extracted into preweighed petri dishes. They were immediately weighed to record the wet weight of the lungs. Then lungs were placed in a dry incubator at 60°C for 7 days, after which time they were weighed again to record the dry weight of the lungs. The weight of the petri dish was subtracted to calculate both the wet and dry lung weights. The ratio of wet to dry lung weight was used to determine the pulmonary edema in the infected lungs.

### Serum complete blood count and lactate dehydrogenase measurements.

Mice were sacrificed at 7 to 9 dpi. Blood was collected by cardiac puncture into 1.1 mL Z-Gel Micro tubes (catalog no. 41.1378.005; Sarstedt). CBC with automated differential was performed at the UGA Athens Veterinary Diagnostic Laboratory. Lactate dehydrogenase (LDH) was measured with a QuantiChrom lactate dehydrogenase kit (catalog no. D2DH-100; VWR) using 3 μL of serum.

### Statistical analysis.

*P* values were calculated with GraphPad Prism software version 9.2.0.332 using Student’s *t* test or one-way analysis of variance (ANOVA) with Tukey’s multiple-comparison test, depending on the number of groups being compared. Differences were considered statistically significant if *P* values were *≤*0.05. Data represent means ± standard error of the mean (SEM).
